# Surgical strategy, methods of reconstruction, surgical margins and postoperative complications in oncoplastic breast surgery

**DOI:** 10.1007/s00238-013-0922-4

**Published:** 2014-02-01

**Authors:** Michael Rose, Jonas Manjer, Anita Ringberg, Henry Svensson

**Affiliations:** 1 Department of Surgery, Section of Plastic Surgery, Sydvestjysk Sygehus, Esbjerg, Denmark; 2 Department of Plastic Surgery, Aleris-Hamlet Hospitaler, Copenhagen, Denmark; 3 Department of Clinical Sciences in Malmö, Lund University, Lund, Sweden; 4 Department of Plastic and Reconstructive Surgery, Skåne University Hospital, Malmö, Sweden; 5 Kirurgisk Klinik, Plastikirurgisk Sektion, Sydvestjysk Sygehus, Finsensgade 35, 6700 Esbjerg, Denmark

**Keywords:** Breast cancer, Oncoplastic surgery, Methods of reconstruction, Surgical margins, Postoperative complications, Partial breast reconstruction

## Abstract

**Background:**

Oncoplastic breast surgery is an evolving discipline in the surgical treatment of breast cancer aimed to improve the outcome.

**Methods:**

Oncoplastic breast surgery was performed between January 2008 and December 2010 on 72 women with 74 breast cancers selected from a population of 1,018 primary breast cancer patients. Careful preoperative planning revealed the possibility of partial breast reconstruction with volume reduction, volume displacement or volume replacement depending on breast size as well as tumour size and location. Data were registered consecutively.

**Results:**

The surgical plan was successful in all but one case, where a mastectomy had to be performed during the primary surgery. In 53 cases, a contralateral mammoplasty was performed during the operation to achieve symmetry. During the follow-up period until November 2011, only one patient needed corrective surgery. Final histopathological examination indicated that seven cases required extended resection and three cases required a mastectomy. Five patients experienced delayed wound healing, although complications requiring further surgery occurred for the reconstructed breast in four cases, the contralateral breast in three cases and the axilla after exaeresis in two cases because of haematoma. Such complications led to slight delay in adjuvant therapy for four patients.

**Conclusions:**

This study demonstrates that it is feasible to implement oncoplastic breast surgery into daily clinical practice as a supplement to conventional breast cancer surgery. As such, oncoplastic breast surgery may provide a markedly better outcome than breast-conserving surgery in terms of shape and symmetry without compromising the surgical margins.

Level of Evidence: Level IV, prognostic/risk study.

## Introduction

In recent years, the discipline or subspecialty of oncoplastic breast surgery has grown considerably as breast and plastic surgeons increasingly work together to surgically treat breast cancer patients [1–10]. The reasoning behind oncoplastic breast surgery is twofold: to ensure that patients are treated with radical cancer surgery and to achieve the best possible cosmetic and resilient result, including a naturally shaped breast with acceptable symmetry [8–16]. Oncoplastic breast surgery involves partial breast reconstruction and, if indicated, contralateral surgery all in one surgical procedure. Secondary reconstructive procedures are rarely required.

As a breast cancer treatment, oncoplastic breast surgery is expected to be similar to conventional breast-conserving surgery with regards to resection margins, locoregional recurrence and metastatic disease [6, 17–21]. Nevertheless, it should be noted that specific studies investigating longterm safety concerning locoregional recurrence and metastatic disease after oncoplastic breast surgery are limited. Cancer surveillance after partial breast reconstruction seems not to be impaired [22].

As oncoplastic breast surgery is often more complex and involves both breasts, the occurrence of early postoperative complications could be expected to be higher than the occurrence of complications after breast-conserving surgery [18]. The primary concern for postoperative complications is the potential delay in commencement of adjuvant therapy. In a recent review by McIntosh and O’Donogue, they find that the available data were inadequate to conclude whether or not complications after oncoplastic breast surgery delay delivery of adjuvant therapy compared with conventional breast-conserving surgery [18].

Oncoplastic breast surgery is the application of surgical techniques from conventional breast-conserving surgery as well as plastic and reconstructive surgery, resulting in an improved aesthetic outcome for breast cancer patients. Thus, oncoplastic breast surgery may reduce the number of overall breast cancer patients undergoing a mastectomy [4, 5, 11, 15, 21].

Previous studies have shown that, compared with mastectomy, breast-conserving surgery has less of an impact on a patient’s body image, as well as psychosocial and social aspects of life [13, 15, 23]. By providing a better aesthetic outcome than tumour resection alone, oncoplastic breast surgery may also improve quality of life for breast cancer patients after surgical treatment [1, 3, 13].

Several factors, such as tumour size, tumour location and size of the affected breast, need to be addressed when considering oncoplastic surgery for partial breast reconstruction [3, 4, 11, 14–16, 21]. Furthermore, the choice of reconstruction method determines the need for surgery on the contralateral breast to ensure symmetry. Patient preference for a particular method of reconstruction and the sentiment towards possible contralateral surgery must also be considered [14, 16, 21].

Implementation of oncoplastic breast surgery into daily clinical practice will undoubtedly take time [1, 2]. Our team of plastic and reconstructive surgeons and breast surgeons continues to develop and perfect surgical techniques since routine oncoplastic breast surgery was introduced at our practice. Successful implementation also involves learning to determine which patients are suitable for oncoplastic breast surgery and to find the most suitable technique of oncoplastic breast surgery in each patient.

In the current study, we present and evaluate our strategy in the surgical planning of oncoplastic breast surgery in terms of different reconstructions methods related to tumour size, tumour location and size of the breast. Furthermore, we present and evaluate results of surgical radicality in terms of resection margins, surgery due to insufficient resection margins and late positive sentinel nodes and early postoperative complications.

## Materials and methods

### Setting

In January 2008, oncoplastic breast surgery was introduced for selected patients with primary breast cancer. The surgical team consisted of breast surgeons as well as plastic and reconstructive surgeons from the public hospital Sydvest Sygehus, Esbjerg, DK, Surgical Department, Section of Breast Surgery and Section of Plastic and Reconstructive Surgery, as well as the private hospital Privathospitalet Aleris-Hamlet, Copenhagen, DK, Department of Breast and Plastic Surgery. In October 2010, oncoplastic breast surgery was also introduced at the public hospital Sygehus Soenderjylland, Aabenraa, DK, Department of Surgery, Section of Breast Surgery.

The two public hospitals recruited their patients from their uptake areas whereas patients actively chose the private hospital regardless of place of residence.

A team of five plastic surgeons and two breast surgeons performed the surgery at the public hospitals, while a team of two plastic surgeons and two breast surgeons performed the surgery at the private hospital.

### Patient selection

Patients were diagnosed with primary breast cancer by clinical mammography and biopsy. They were examined by a breast surgeon. If conventional breast-conserving surgery was predicted to result in an unfavourable cosmetic outcome, or if mastectomy was the primary recommendation based on tumour size and location rather than for oncological reasons, then the patient was informed of the alternative of oncoplastic breast surgery. Potential candidates for oncoplastic breast surgery were hence referred to a plastic and reconstructive surgeon for additional consultation to assess possible reconstruction methods. If oncoplastic breast surgery was deemed suitable, planning for immediate partial reconstruction and optional contralateral surgery to ensure symmetry ensued.

Patients for whom radiation therapy was contraindicated as well as patients with multicentric tumours or extensive ductal carcinoma in situ (DCIS) were excluded. Known systemic dissemination of the cancer was regarded as a relative contraindication. Age, comorbidity, smoking, alcohol habits and overweight status were not considered contraindications for oncoplastic breast surgery.

## Patients

Patients were selected from the general population of breast cancer patients who were scheduled to have surgery between January 2008 and December 2010 (Table 1). Oncoplastic breast surgery was performed on 72 patients with a total 74 primary breast carcinomas (two cases with bilateral breast cancer). Oncoplastic breast surgery was performed on 44 patients at Sydvestjysk Sygehus, 23 patients at Aleris-Hamlet Privathospitaler and 5 patients at Sygehus Soenderjylland. Nineteen patients were referred from the mammography screening programme, whereas 53 patients were referred by general practitioners or others specialists after detection of a palpable breast tumour or a mammography showing areas of suspected malignancy for further evaluation.

**Table 1:** Summary of breast cancer-related surgeries for patients treated at Sydvestjysk Sygehus (Esbjerg) and Privathospitalet Aleris-Hamlet (Copenhagen) between January 2008 and December 2010, as well as breast cancer patients treated at Sygehus Soenderjylland (Aabenraa) from October 2010 to December 2010

	Sydvestjysk Sygehus	Privathospitalet Aleris-Hamlet	Sygehus Soenderjylland
	Esbjerg	Copenhagen	Aabenraa
No. of patients	736	115	95
Mastectomy	283 (38 %)	45 (39 %)	37 (39 %)
Partial mastectomy	409 (56 %)	47 (41 %)	53 (61 %)
Oncoplastic surgery	44 (6 %)	23 (20 %)	5 (5.2 %)

The mean age was 53, ranging between 31 and 69 years of age. Invasive ductal carcinomas comprised 87 % of tumours, while 3 % were invasive lobular carcinomas, 6 % were mixed types and 4 % were DCIS. Mean tumour size was 21 mm, ranging from 6 to 50 mm. None of the 45 patients with contralateral reduction mammoplasty were determined to have invasive or in situ carcinoma in the resected breast tissue.

Until November 2011, the median observation period was 26 months, ranging from 11 to 46 months. During this time, one patient experienced local recurrence of a metaplastic carcinoma while undergoing chemotherapy and another patient died from metastatic spread of the disease. One patient had surgery during the observation period to correct for breast asymmetry after radiation therapy. The correction was done as a re-reduction mammoplasty in the contralateral breast.

## Surgical strategy

### Reconstruction techniques

To obtain a reconstructed breast with a natural shape and acceptable symmetry to the contralateral breast, volume reduction, volume displacement and volume replacement techniques were utilised [9, 12, 14–16, 24–28]. The volume reduction technique involves tumour resection along with the normal tissue resected in a reduction mammoplasty. Therefore, the partial mastectomy is integrated into the reduction mammoplasty procedure. Conversely, in the volume displacement technique, the defect after partial mastectomy is filled with internal flaps of breast tissue, whereas the replacement technique involves filling the defect with external flaps of tissue from outside the breast on the thoracic wall. When the reconstruction was done using the volume reduction or volume displacement technique, reduction mammoplasty or mastopexy was simultaneously performed on the contralateral breast to ensure symmetry.

### Tumour size, tumour location and breast size

Several factors, such as tumour size, tumour location, size of the affected breast and size of the contralateral breast, need to be considered when planning oncoplastic breast surgery. Breast size was categorised as small, medium or large. When using these terms we do not refer to absolute measurements in volume or weight. The terms rather refer to a clinical judgment of the relation between the size and location of the tumour on one hand, and the size of the breast on the other. This interrelation determines the expected defect after tumour resection related to the residual breast volume, and consequently the feasible techniques for reconstruction. As a clinical norm a small breast is up to 250 cm^3^, a medium one 250–500 cm^3^ and a large one 500 cm^3^ or more. Typically, a small breast requires reconstruction using volume replacement techniques, where extra mammary tissue is utilised. Reconstruction by volume replacement may also be an option for medium or large sized breasts. However, a medium to large breast is more suited for reconstruction using volume reduction or volume displacement techniques.

**Fig. 1:**
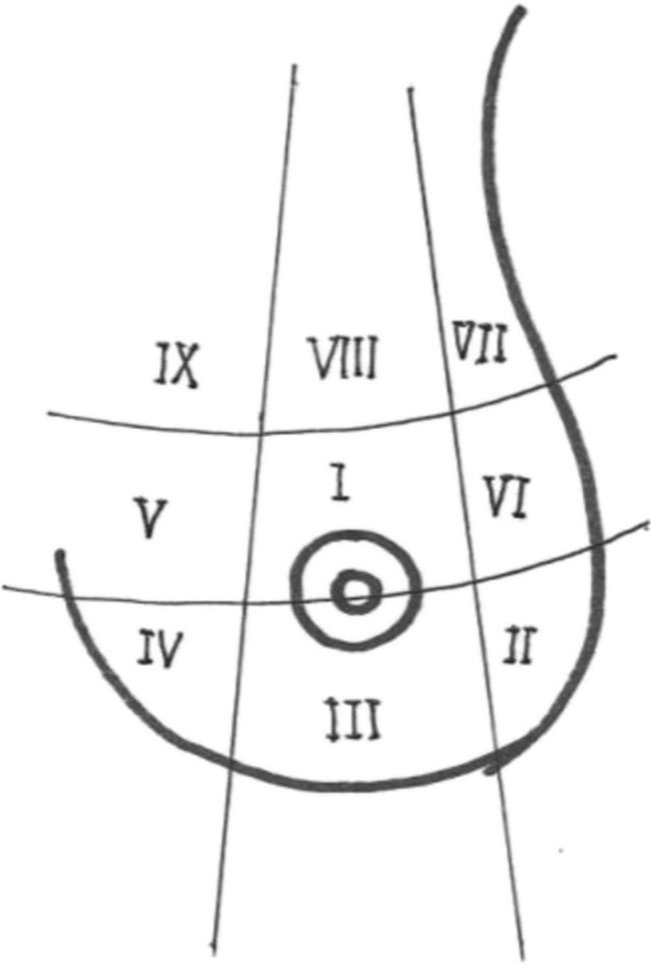
Illustration of the distribution of zones in the breast described by McCulley and MacMillan [14]

Tumour size itself does not have any impact on the decision to perform an immediate partial reconstruction. Rather, the size of the tumour relative to the affected breast, that is the size of the defect after tumour resection in relation to the size of the breast, determines if an immediate partial reconstruction is feasible. If so, the location of the tumour also has to be taken into consideration when deciding which method of reconstruction to recommend. As guidance for planning and evaluation of the reconstruction we used tumour location in zones I–IX described by McCulley and Macmillan [12, 14] as shown in Fig. 1.

### Surgical planning

In addition to tumour size, tumour location and breast size, the patient’s acceptance of the recommended reconstruction method, including potential donor sites and the possibility of contralateral breast surgery to ensure symmetry, was factored into the planning process for immediate partial reconstruction.

Patients were divided into four groups to facilitate surgical planning and postoperative evaluation. Group I included patients with medium to large breasts and tumours located in zones II–IV, whereas group II consisted of patients with medium to large breasts and tumours located in zones V–IX. Group III patients possessed medium to large breasts and tumours in zone I. Finally, group IV consisted of patients with small breasts and tumours in zones I–IX.

### Group I: lower region, medium to large breasts (zones II–IV)

**Fig. 2:**
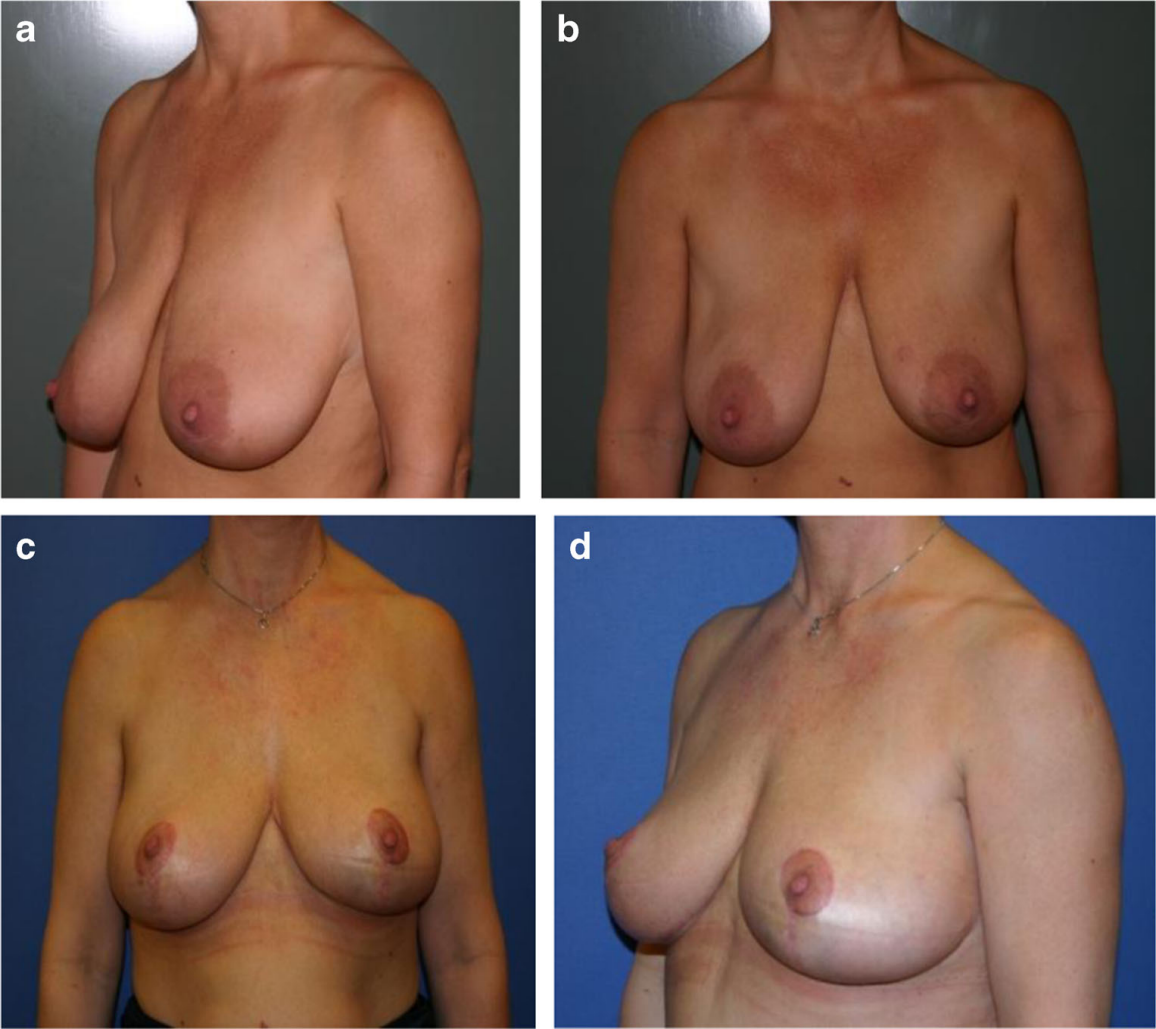
Patient with medium to large breasts from group I. A 42-year-old woman with a 15-mm invasive ductal carcinoma located in the lower central region of the left breast (zone III). Weight of lumpectomy at 80 g, reconstruction with volume reduction technique and contralateral reduction mammoplasty. Preoperative photos (**a**, **b**) and results after radiotherapy 2 years post-surgery (**c**, **d**)

In patients with tumours located in the lower lateral, lower central or lower medial regions (zones II–IV) and medium to large breasts, the resection and reconstruction was planned as a volume reduction as part of a reduction mammoplasty as shown in Fig. 2 [14]. The tumour resection could often be performed as an en bloc resection with very wide margins. To ensure symmetry, a contralateral reduction mammoplasty was performed simultaneously with resection of the same amount of tissue as in the reconstructed breast.

### Group II: upper or mid region, medium to large breasts (zones V–IX)

**Fig. 3:**
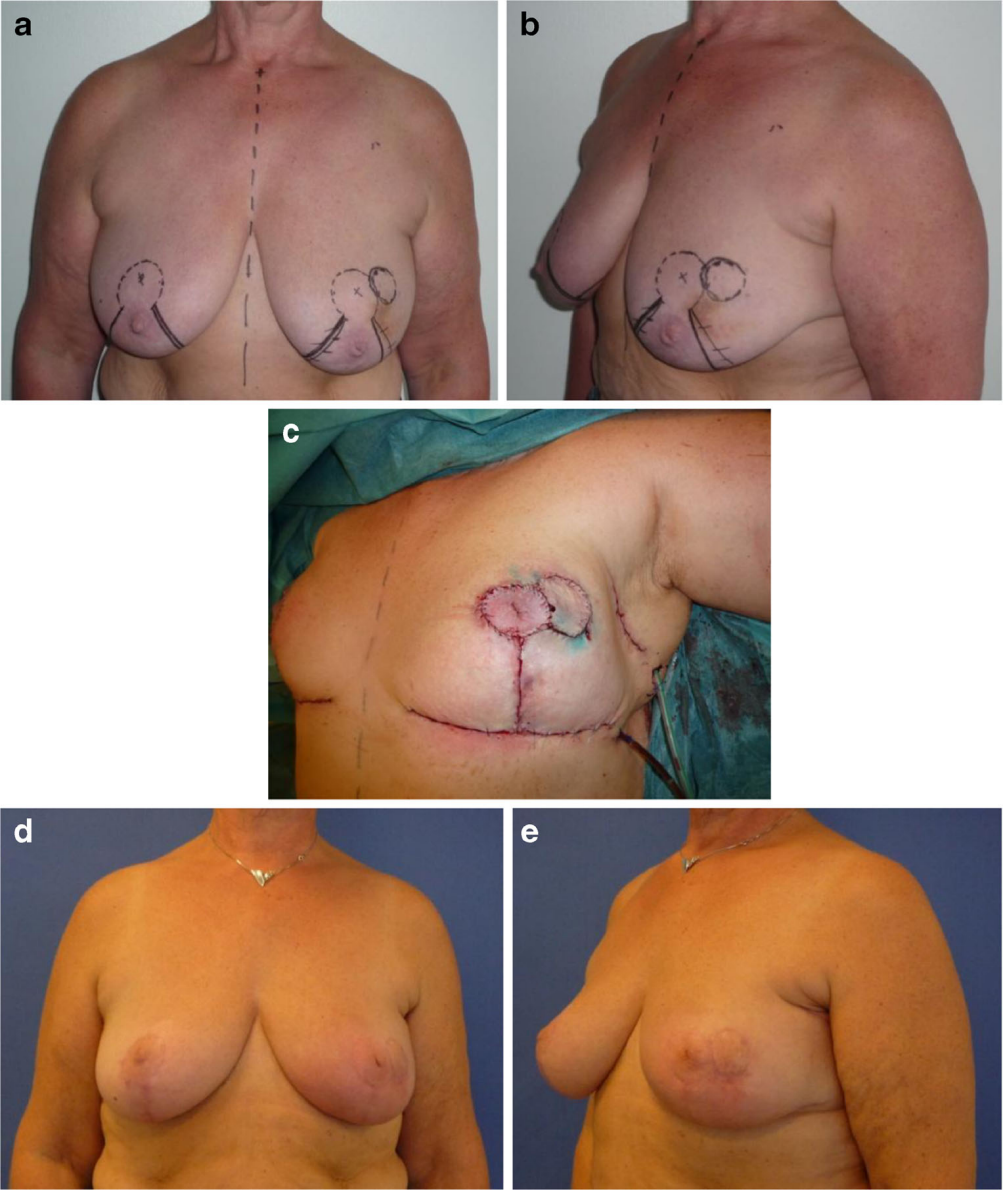
Patient with medium to large breasts from group II. A 59-year-old woman with a 17-mm invasive ductal carcinoma in the upper region (zone VII) of the left breast. Weight of lumpectomy at 41 g, reconstruction with volume displacement technique with inferior-based extended flap with skin island and contralateral reduction mammoplasty. Photos preoperative (**a**, **b**), peroperative (**c**) and 3 months postoperative (**d**, **e**)

If tumours were located in the mid or upper region of the breast (zones V–IX), resection was not possible within a conventional reduction mammoplasty. In patients with medium to large breasts, the reconstruction was planned as a volume displacement of residual breast tissue (Fig. 3). Most of these reconstructions were performed as part of a simultaneous reduction mammoplasty. All patients received reduction mammoplasty of the contralateral breast to ensure symmetry. Some patients with medium sized breasts who declined surgery on the contralateral breast were reconstructed using *volume replacement*, where the volume of the reconstructed breast was not altered and contralateral surgery could be avoided.

### Group III: central tumours, medium to large breasts (zone I)

**Fig. 4:**
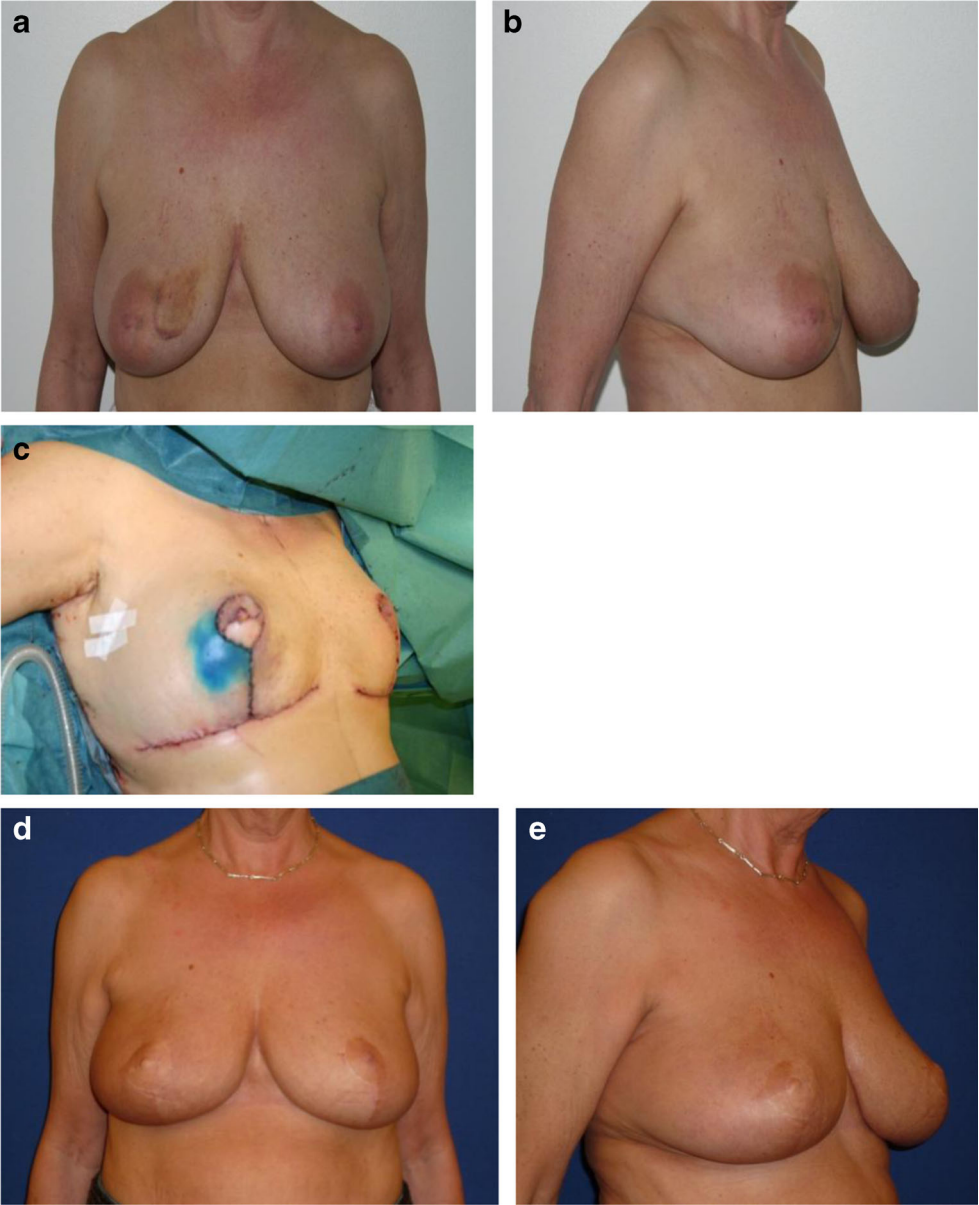
Patient with medium to large breasts from group III. A 63-year-old woman with a 19-mm invasive ductal carcinoma located in the central region of the right breast (zone I). Weight of lumpectomy at 124 g, which included the nipple-areola complex, reconstruction with volume displacement technique with inferior-based flap and immediate nipple reconstruction and contralateral reduction mammoplasty. Photos taken preoperative (**a**, **b**), peroperative (**c**) and 2 years postoperative (**d**, **e**)

Tumour resection of a central tumour (zone I) involved resection of tissue, which is not typically included in a regular reduction mammoplasty. For some patients, it included either the entire or part of the nipple-areola complex (NAC). For patients with medium or large breasts, the reconstruction was planned as a *volume displacement* of residual breast tissue and a contralateral reduction mammoplasty (Fig. 4). If tumour resection included the NAC, nipple reconstruction was performed at the same time or as a delayed procedure.

### Group IV: small breast (zones I–IX)

**Fig. 5:**
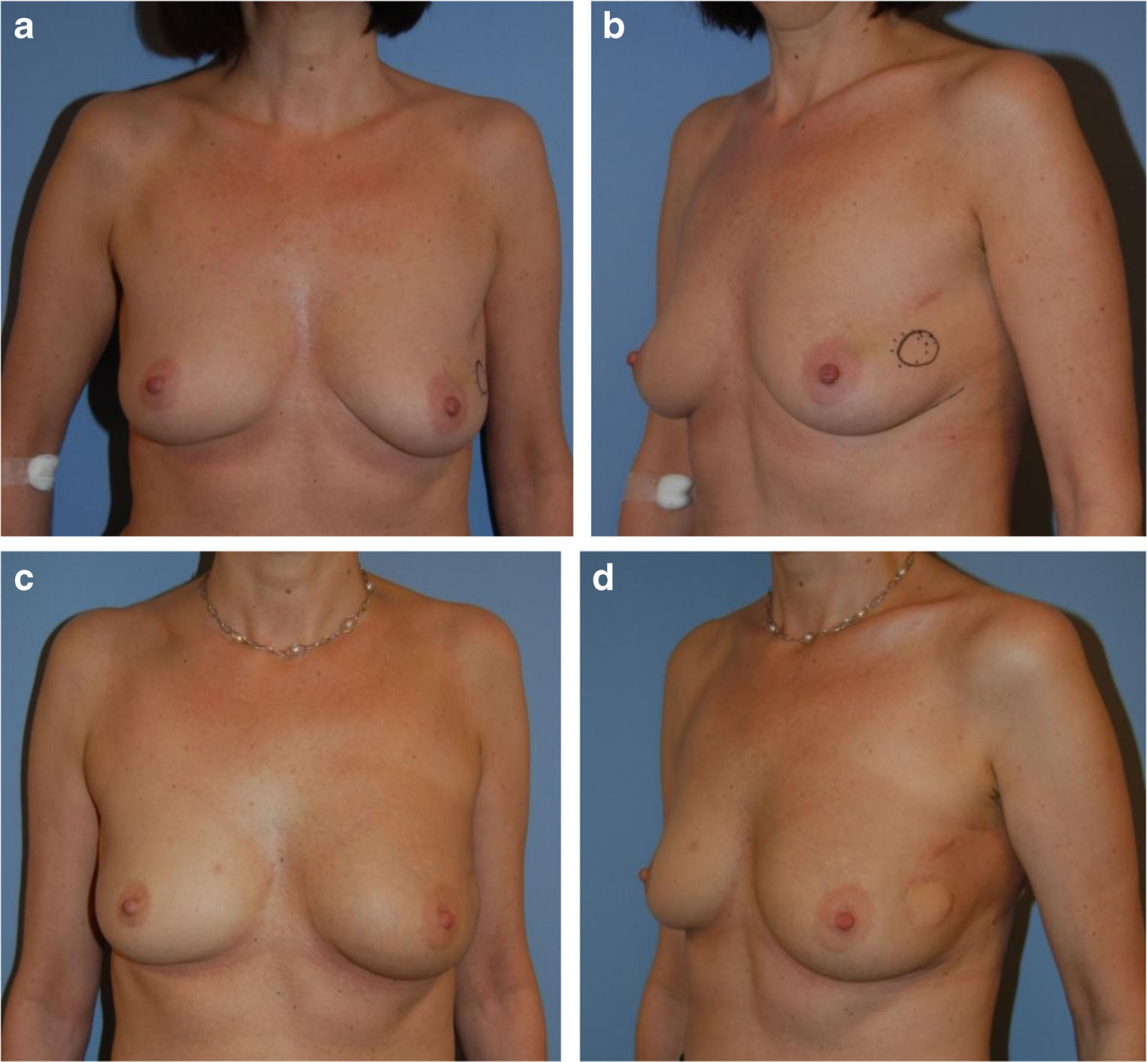
Patient with small breasts from group IV. A 46-year-old woman with a 25-mm invasive ductal carcinoma in the lateral region (zone VI) of the left breast. Weight of lumpectomy at 40 g, reconstruction with volume replacement technique with tunnelled thoracodorsal flap with skin island and no contralateral surgery. Pre- (**a**, **b**) and postoperative photos after 3 months (**c**, **d**)

Regardless of tumour location, reconstruction with volume reduction or volume displacement is not an option for patients with small breasts because no residual breast tissue would be available for these techniques after tumour resection. As such, reconstruction after tumour resection was planned as a *volume replacement* using external flaps. These patients were planned for reconstruction with a tunnelled lateral thoracodorsal flap with skin island [26], a TAP flap [27] or a muscle-sparing latissimus dorsi flap [28]. Using these methods, the reconstructed breast maintained the preoperative size and generally no contralateral surgery was needed (Fig. 5). However, one patient had a contralateral mastopexy to relocate the position of the NAC.

### Peroperative management

Surgery was performed by a team of breast surgeons working closely with plastic and reconstructive surgeons. The responsibilities and tasks of each surgeon were clearly defined before surgery. The breast surgeon was responsible for the ablative procedure, including tumour resection and sentinel node biopsy. If a sentinel node biopsy indicated metastasis or if metastasis to the axilla was diagnosed preoperatively, the breast surgeon also performed axillary exaeresis. The plastic surgeon was responsible for reconstruction and contralateral reduction mammoplasty or mastopexy. If a contralateral procedure was required, the operation was performed simultaneously with the ablative procedures to minimise time in the operation room.

Resected tissue was peroperatively examined macroscopically by a pathologist to ensure free margins. Later, the specimen was evaluated by routine histological methods. The tumour cavity was marked by metal clips for the orientation of radiation therapy. In order to avoid contamination, the operation fields were kept strictly separated in cancer and non-cancer areas including the use of separate instruments.

### Postoperative management

During the postoperative period, patients recovered as in-patients at the Department of Breast Surgery and were usually discharged after 2–5 days. Post-surgery patients were referred to adjuvant therapy according national guidelines by the Danish Breast Cancer Group (DBCG) [29]. Patients were seen by a plastic surgeon for follow-up visits at 3, 12 and 24 months post-surgery.

## Results

### Surgical strategy

Surgery was performed as planned with tumour resection, immediate partial reconstruction and, if needed, contralateral surgery to ensure symmetry in 73 of 74 procedures. However, in one patient, the planned breast-conserving procedure had to be converted to a mastectomy to ensure free margins, due to DCIS extending more than 50 mm (Table 3). For this patient, reconstructive surgery using a breast implant was performed at a later date.

The distribution of patients in groups I to IV, as well as the methods of reconstruction used for each group, are shown in Table 2. Tumour resections in group I had a mean specimen weight of 367 g, ranging from 41 to 1,630 g, which was considerably larger than tumour resections in group II, III and IV. The mean weight in group II was 138 g (range from 27 to 463 g), whereas the mean specimen weight in patients with central tumours in group III was 83 g (ranged from 27 to 124 g). Group IV patients with small breasts had an average resected tumour weight of 55 g, ranging from 20 to 127 g.

**Table 2:** Tumour resections with immediate partial breast reconstruction in relation to tumour location (zones) and size of the breast (small, medium and large), weight of resection (grammes), methods of reconstruction and contralateral surgery group I–IV

Group:	No.	Tumour resection weight mean (range)	Reconstruction method	No.	Contralateral surgery
I (medium to large, zones II–IV)	17	367 g (41–1,630)	Superior flaps	17	17/17 (100 %)
II (medium to large, zones V–IX)	25	138 g (27–463)	Inferior flaps	16	22/25 (88 %)
			Superior flaps	4	
			Lateral flaps	1	
			Rotation flap	1	
			Tunnelled LT flap^a^	2	
			TAP flap	1	
III (medium to large, zone l)	14	83 g (27–124)	Inferior flaps	13	14/14 (100 %)
			Superior flap	1	
IV (small breasts, zones I–IX)	17	55 g (20–127)	Tunnelled LT flap^a^	13	1/17 (6 %)
			LD flap	3	
			Rotation flap+LT	1	
Total (groups I–IV, zones I–IX)	73	157 (20–1630)			54/73 (74 %)

For 1 of 72 patients, mastectomy was required instead of oncoplastic surgery due to non-radical lumpectomy due to tumour histology

*LT* lateral thoracodorsal flap; *LD* muscle-sparing latissimus dorsi flap

^a^Tunneled lateral fasciocutan thoracodorsal flap with skin island

As summarised in Table 2, reconstructions in group I were all performed using a volume reduction technique with superior flaps as part of a reduction mammoplasty. Reconstructions in group II patients with medium to large breasts were done using either a volume displacement (22/25) or volume replacement (3/25) technique involving several different types of flaps, mostly extended or secondary pedicled flaps. All patients with central tumours in group III (*n* = 14) had reconstructions performed using the displacement technique. Similarly, all patients with small breasts in group IV (*n* = 17) were had reconstructions using a volume replacement technique with different types of external flaps. Overall, 54 patients (74 %) elected for surgery on the contralateral breast to ensure symmetry using either a reduction mammoplasty (45/54) or mastopexy (9/54). Nineteen of 73 patients, including all patients with small breasts, did not require contralateral breast surgery since all 19 were reconstructed using a volume replacement technique.

### Surgical radicality

In 73 of 74 resections, the tumour was resected with free margins based on peroperative macroscopic evaluation during the oncoplastic procedures (Table 3). For ten cases (14 %), peroperative macroscopic evaluation was corrected after postoperative histological evaluation. In seven cases (10 %), free margins were achieved by re-resection; whereas three cases (4 %) required a mastectomy (Table 3).

**Table 3:** Primary mastectomies, primary sufficient resection margins, primary axillary exaeresis, secondary mastectomies, re-resections and secondary axillary exaeresis after final pathological evaluation

	No. of breast	Percent
Primary surgery		
Mastectomy	1	1.4
Sufficient resection margins peroperative	73	98.6
Peroperative positive sentinel node + axillary exaeresis	38	51.4
Secondary surgery		
Insufficient resection margins in final pathological evaluation causing mastectomies	3	4.0
Insufficient resection margins in final pathological evaluation causing re-resection	7	9.5
Late positive sentinel node causing secondary axillary exaeresis	9	12.1

*n* = 74; where *n* is the numbers of breasts with cancer. Two patients had bilateral cancers

For 38 patients (51 %), metastasis to axillary lymph nodes was determined preoperatively or peroperatively after sentinel node biopsy. Axillary exaeresis was performed at the time of primary surgery (Table 3). An additional nine patients (12 %) had positive sentinel node biopsy after postoperative microscopic evaluation. Axillary exaeresis was performed at a later date according to national guidelines by the DBCG [29] (Table 4).

**Table 4:** Secondary surgery in general anaesthesia due to complications and local disease control

Complication	Site of complication	No	Percent
Haematoma	Reconstruction	4	(5.4 %)
	Contralateral breast	3	(4.1 %)
	Axilla	2	(2.7 %)
Disease control			
Re-resections		7	(9.5 %)
Axillary exaeresis due to late positive SN		9	(12.3 %)
Mastectomy		3	(4.1 %)
Reoperations total		28	(38.3 %)

*n* = 73. One patient had a haematoma on both the reconstructed and contralateral breast after reduction mammoplasty, as well as a third haematoma after re-resection because of insufficient resection margins

### Surgery due to insufficient primary surgery or postoperative complications

Subsequent surgery performed under general anaesthesia after primary tumour resection and reconstructive surgery was necessary due to two scenarios. One scenario was insufficient resection margins or positive sentinel node biopsy after final microscopic evaluation in 19 patients (26 %) and the second scenario was postoperative complications in 9 patients (12 %) (Table 4). Postoperative complications occurred in the reconstructed breast, the contralateral breast or the axilla when axillary exaeresis had been performed (Table 5). Haematoma was the most common complication, which occurs nine times in seven different patients (10 %).

**Table 5:** Summary of complications observed after oncoplastic surgery

	Haematoma	Necrosis	Seroma	Delayed wound healing	Infection
Reconstructed breast (*n* = 73)	4 (5.5 %)	1	0	3	0
Donor site (*n* = 20)	0 (0.0 %)	0	0	0	0
Contralateral breast (*n* = 53)	3 (5.6 %)	0	0	2	0
Axilla (*n* = 45)	2 (4.4 %)	0	1	0	1
Total	9	1	1	5	1

One patient had a haematoma on both the reconstructed and contralateral breast after reduction mammoplasty, whereas another patient experienced delayed wound healing (more than 4 weeks) on both breasts

Adjuvant therapy is according to the DBCG guidelines planned to begin 4 weeks postoperatively [29]. When complications led to onset beyond 4 weeks the onset was regarded as delayed. Four patients (6 %) had delayed onset of adjuvant therapy due to delayed wound healing. The delay was 13, 14, 24 and 50 days respectively.

## Discussion

The surgical strategy for oncoplastic breast surgery strives for radical cancer surgery as well as a good cosmetic and resilient outcome and by this improving the outcome of breast cancer surgery. The purpose of the current study was to present an evaluation of a surgical strategy pertinent to a wide range of variations in tumour size and location and breast size.

Patients included in the study were comparable to patients in previous studies examining similar aspects of conventional breast-conserving surgery with regards to tumour type, location and size [11–14, 16–18, 21]. The study cohort included patients with small to large breasts and tumours of various sizes and locations within the breast, reflecting the general variations in breast cancer patients.

The surgical strategy was designed to meet the reconstructive challenges presented in the whole population of breast cancer patients. Multiple methods of reconstruction were used, including volume reduction, volume displacement and volume replacement techniques, demonstrating that surgeons performing oncoplastic breast surgery require familiarity and experience with several reconstructive techniques [9, 12–16, 21, 24–28].

If the reconstructed breast was predicted to be markedly smaller or less ptotic than the contralateral breast, the patient was offered a contralateral reduction mammaplasty or mastopexy, which are routinely performed in a many hospitals [8, 9, 11–16, 20]. It is debated whether or not postoperative radiation therapy cause subsequent shrinkage due to fibrosis or chronic oedema leading to an increase in breast volume. Regardless, both situations would make immediate contralateral surgery inappropriate [8, 9, 30]. During our observation period of more than 2 years, on average, a supplemental corrective procedure had to be done just for one patient. This supports the use of immediate contralateral surgery and is consistent with previous studies [11, 12, 14, 21].

With our strategy, we had positive resection margins in ten of our patients (14 %) for which seven (10 %) required re-resection and three (4 %) required mastectomy. This result is equal to or better than figures reported in previous studies [11, 12, 17, 18, 21, 31, 32].

One would think that oncoplastic breast surgery with immediate reconstruction involving internal or external flaps and bilateral surgery would be followed by an increase in postoperative complications compared with unilateral breast-conserving surgery. With our strategy we noted haematomas requiring surgery in 12 % of cases, of which 5 % were located in the reconstructed breast, 4 % in the contralateral breast and 3 % in the axilla. No flap necrosis was observed. Results are consistent with previously reported rates of early postoperative complications [11, 12, 18].

Administration of adjuvant therapy is important for the optimal treatment of breast cancer. As such, it is important that complications after surgery do not delay delivery of adjuvant therapy. Delay of adjuvant therapy occurred in 4 of our patients (6 %) due to wound healing problems, which have also been reported in previous studies [9, 11, 12, 18]. Thus, complications after oncoplastic breast surgery have little negative impact on the timely administration of adjuvant therapy.

In the realm of our study, 1,018 women had surgery due to breast cancer. Sixty-two percent had breast-conserving surgery where 7 % represent oncoplastic surgery (Table 1). The low percentage of patients operated with oncoplastic surgery indicates that far from all patients that would be suitable for oncoplastic surgery are presented for this option. This view is supported by Urban et al. [6] and Baildam [7] who reported that up to 30 % of patients treated by breast-conserving surgery experience deformities that require complementary surgery. To increase the number of oncoplastic breast procedures, the capacity of the breast surgeon to anticipate various reconstruction methods is essential and underlines the necessity of team work between breast surgeons and plastic and reconstructive surgeons.

Without the option of oncoplastic surgery, most patients with tumours in small breasts, and some of the patients with central tumours would be referred to a mastectomy and eventually an immediate or a delayed reconstruction [11, 16]. Especially in these circumstances, our strategy with the use of external flaps becomes particularly relevant.

In conclusion, it is possible and safe to carry through a preoperatively planned method for immediate partial breast reconstruction in a wide range of variations in tumour size, tumour location and breast size, and this demonstrates the feasibility to implement oncoplastic breast surgery into daily clinical practice based on our strategy.
